# Calcium Hydroxylapatite (CaHA) and Aesthetic Outcomes: A Systematic Review of Controlled Clinical Trials

**DOI:** 10.3390/jcm13061686

**Published:** 2024-03-14

**Authors:** Mojgan Amiri, Renald Meçani, Erand Llanaj, Christa D. Niehot, Terri L. Phillips, Katherine Goldie, Janina Kolb, Taulant Muka, Hua Daughtry

**Affiliations:** 1 Epistudia, 3008 Bern, Switzerland; mojgan.amiri@epistudia.com (M.A.);; 2 Department of Endocrinology and Diabetology, Medical University of Graz, 8010 Graz, Austria; 3 Department of Molecular Epidemiology, German Institute of Human Nutrition Potsdam-Rehbrücke, 14558 Nuthetal, Germany; erand.llanaj@dife.de; 4 German Center for Diabetes Research (DZD), 85764 München-Neuherberg, Germany; 5 Literature Searches Support, 3314 SC Dordrecht, The Netherlands; literature.searches.supp@gmail.com; 6 Merz North America, Inc., Raleigh, NC 27615, USA; terri.phillips@merz.com (T.L.P.); carina.daughtry@merz.com (H.D.); 7 Clinic 77, London W1G 9QD, UK; kate@clinic77.co.uk; 8 Merz Aesthetics GmbH, 60318 Frankfurt am Main, Germany; janina.kolb@merz.de

**Keywords:** systematic review, calcium hydroxylapatite (CaHA), effectiveness, aesthetic improvement

## Abstract

**Background**: This study aimed to systematically review and summarize the available controlled clinical trials on the effectiveness of calcium hydroxylapatite (CaHA) in terms of aesthetic outcomes, skin-aging-related outcomes, and patient/investigator satisfaction. **Methods**: We included controlled clinical trials involving at least 10 human adults that examined the effects of CaHA on aesthetic and skin-aging-related outcomes and satisfaction. Due to the high heterogeneity among the included studies, only a qualitative analysis is provided. **Results**: Out of 2935 relevant references, 13 studies were included, of which 8 studies focused on facial areas and 5 on dorsum of hand. CaHA injection was associated with enhancements in global aesthetic improvement scale, whether applied in facial regions or on the dorsum of hands. The findings suggested high patients’ satisfaction following CaHA when applied to facial areas. Studies highlighted improvements in hand grading scales and a reduction in facial wrinkles. **Conclusions**: Current evidence suggests that CaHA injections improve aesthetic results, including facial areas, such as nasolabial folds and jawline, and hands, with high levels of satisfaction. Considering the methodological limitations and heterogeneous comparisons groups, additional controlled clinical trials would contribute to a better understanding of the applications and advantages offered by CaHA.

## 1. Introduction

The objective of aesthetic medicine is directed towards addressing the visible manifestations of skin aging, resulting from immunological reactions to tissue degeneration and injury, as well as the body’s efforts to promote tissue repair. Consequently, aesthetic professionals aim to rejuvenate the structure and functionality of aging tissue by modulating these immunological responses through interventions such as introducing foreign materials like dermal fillers [1].

Dermal fillers are widely used as a minimally invasive aesthetic treatment alternative or supplement to surgery, gaining acceptance from both healthcare providers and patients. The Aesthetic Plastic Surgery National Databank documented dermal fillers as the second-top nonsurgical aesthetic procedure in 2020–2021 [2]. Among available dermal fillers, calcium hydroxylapatite (CaHA) has become a common choice in cosmetic procedures [3]. CaHA is a form of hydroxylapatite microspheres in a carboxymethyl cellulose carrier gel that has been used for correcting moderate-to-severe facial wrinkles and folds, as well as replenishing lost volume.

The impact of CaHA on aesthetic purposes can be explained through various reported mechanisms, including promoting cell proliferation, collagen production, angiogenesis, and the formation of elastic fibers and elastin [4]. The aesthetic effectiveness of CaHA has been investigated in several clinical trials and observational studies that report various degrees of improvement in aesthetic scales [5,6,7,8,9] and levels of patient satisfaction [10,11,12,13,14,15]. The majority of available studies reported a comparison between the aesthetic effects of CaHA following injection versus the baseline values [7,14,15,16,17,18,19,20,21,22], and there are limited comparative clinical data on efficacy of CaHA relative to other categories of existing fillers [5,23,24,25,26,27,28].

Therefore, the objective of our study was to conduct a systematic review and comprehensive synthesis of controlled clinical trials investigating the impact of CaHA on aesthetic outcomes, including aesthetic scores, reduction in wrinkles, and enhancements in skin thickness, along with patient and investigators’ satisfaction. Additionally, this study sought to establish a comparative summary of effectiveness of CaHA in comparison to other existing treatments.

## 2. Materials and Methods

This systematic review adhered to recent systematic review guidelines and the PRISMA reporting standards [29,30]. The study protocol was registered in the OSF Registries on 22 December 2022 (registration DOI: https://doi.org/10.17605/OSF.IO/WY49V).

### 2.1. Data Sources and Search Strategy

An expert research librarian developed the search strategies. Embase.com, Medline ALL (Ovid), Web of Science Core Collection, and Cochrane Central were searched from inception up to October 2022. The first 200 results of Google Scholar were imported. To further identify relevant studies, we retrieved all published reviews to check relevant references. The reference lists of the final included original studies were also manually reviewed. The details of the search strategy and keywords are presented in Table S1.

### 2.2. Eligibility Criteria

We included controlled clinical trials (randomized/nonrandomized) with more than 10 participants involving adults (≥18 years) irrespective of specific participants’ characteristics, such as the rating of the severity of wrinkles, volume or tissue loss, or health condition, that examined the effect of CaHA on aesthetic and skin-aging related outcomes and satisfaction. We excluded case reports, reviews, letters to editors, conference abstracts, and studies conducted in animals, children, or adolescents. Non-English language articles were excluded.

### 2.3. Study Selection and Data Extraction

All titles/abstracts were screened in duplicate by two independent researchers according to the eligibility criteria. Afterward, all provided full texts were similarly reviewed in duplicate. Data from the included studies were extracted based on a predesigned Excel form. The primary extracted information included the first author’s name, study design, publication year, location, number of participants, sex distribution of the population, participants’ health status at study entry, age, follow-up duration, ethnicity, skin type, dermal filler brand, injected area, dilution and dosage, injection depth and tool, outcomes assessment methods, adjustments, and any measure of frequency or association.

### 2.4. Quality Assessment

Quality of the included trials were assessed using Cochrane Collaboration’s Tool Risk of Bias 2 (RoB 2), evaluating biases arising from the randomization process, intended intervention, missing data, measurements of the outcomes, and selection of the reported results [31]. The risk of bias judgments for each domain are “low risk of bias”, “some concerns”, or “high risk of bias”.

### 2.5. Statistical Analysis

As there was considerable variability in subjects, outcomes, control groups, and measurement metrics, we conducted a descriptive synthesis, reporting the magnitude, direction, and significance of the effects observed in each study. In this regard, we created separate tables for the characteristics, key findings, and methodological evaluation of the studies.

## 3. Results

### 3.1. Eligible Studies and Study Characteristics

**Figure 1:**
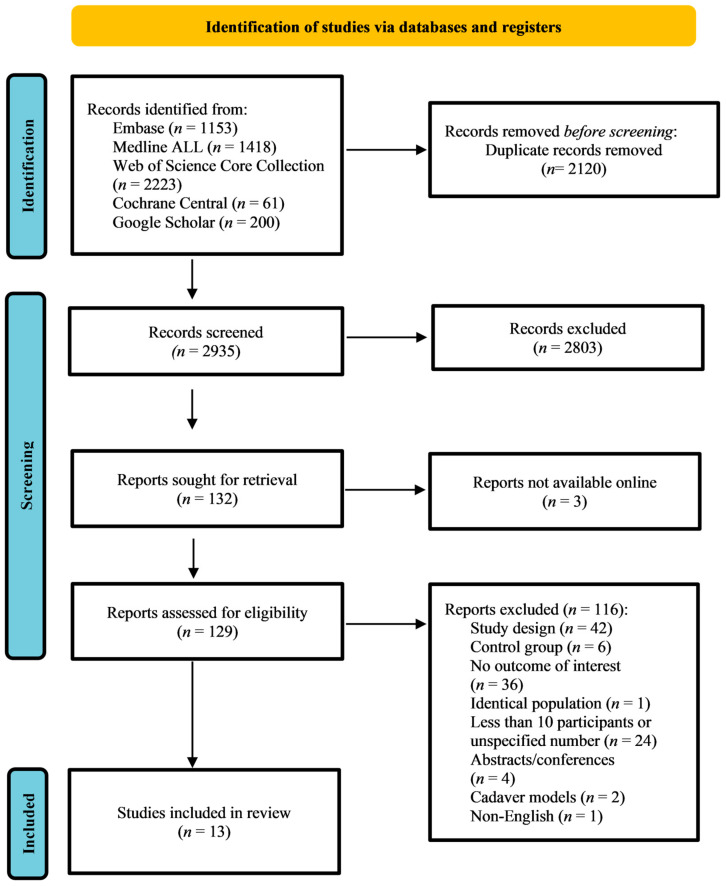
Flowchart of identification, screening, eligibility, inclusion, and exclusion of retrieved studies.

Out of 2935 references, 132 papers were qualified for full-text screening based on predesigned criteria. Of those, 19 controlled clinical trials were retrieved. Among the controlled clinical trials, 6 were excluded since the comparison groups were also intervened with CaHA [11,32,33,34,35,36]. Eventually, 13 papers were included in the final qualitative analysis. Figure 1 outlines the study screening and selection procedure.

The main characteristics of the eligible studies are summarized in Table 1. Studies were published between 2007–2022. A total of 1171 participants were included in this systematic review, and 93.3 percent of the population consisted of females. The mean age of the participants was 50.2 years old, with a standard deviation of 3.93. Among 13 included studies, 8 investigated the effect of CaHA injection on facial areas [5,8,12,27,28,37,38,39] and 5 studies on the dorsum of hands [23,24,25,26,40]. The median of follow-up was 26 weeks (from 4 to 48 weeks) and the sample size ranged between 10 to 194 participants. Four studies were designed as either split-face or hands [5,8,26,38] and the remaining as parallel clinical trials [12,23,24,25,27,28,37,39,40]. In studies focused on facial areas, the comparison treatments of five studies were different types of hyaluronic acids, human-based collagen, poly-L-lactic acid, cross-linked polyacrylamide and fat fillers [5,27,28,38,39]. The control groups of the three remaining studies received no treatment during the trial [8,28,37]. Among studies on dorsum of hands, two used hyaluronic acid as the control treatment [26,40] and the remaining applied no treatment in the control groups [23,24,25]. The most frequently reported outcome was global aesthetic improvement followed by patient satisfaction in facial area and global aesthetic improvement followed by hand grading scales in dorsum of hands. Also, some additional information on assessment tools is provided in Table S4.

### 3.2. Summary of the Quality Assessments

Among the included trials, at least one domain was scored as being at high risk of bias. The intended interventions, randomization process, and outcome measurements were mostly judged as being at high risk of bias or having some concerns. Detailed assessments of risk of bias of the included studies are presented in Table S2.

### 3.3. Summary of the Findings

#### 3.3.1. Facial Area

##### Global Aesthetic Improvement

Five out of eight studies reported the findings global aesthetic improvement scale (GAIS) after CaHA injection in facial areas [5,8,28,37,38]. A split-face clinical trial comparing CaHA with nonanimal-stabilized hyaluronic acid showed a total improvement in GAIS in the CaHA-treated nasolabial folds; evaluators assessed CaHA as superior in 47% of patients and inferior in only 5% compared to the control group (*p* < 0.0001) [5]. Another split-face study examined the effect CaHA and human-based collagen. Investigators reported 20% of patients as very much improved, 40% much improved, 35.7% improved, and 4.3% no change in CaHA-treated area while only 0.9% of patients were evaluated as very much improved, 5.2% much improved, 19.10% improved, 56.5% no change, and 18.3% worse in the control area. No between-groups comparison was reported [38]. A comparison between CaHA and several dermal fillers, including Juvéderm 24, Perlane, and Juvéderm 24 HV, for correction of nasolabial folds showed a statistically significant improvement in GAIS evaluated by investigators in patients receiving CaHA (2% of patients were reported as much improved, 56% as improved, 38% as no change) compared to Juvéderm 24 HV (76% of patients were reported as no change and 24% as worse results; *p* < 0.001) and Perlane (8% as much improved, 40% improved, 51% no change, and 1% worse; *p* = 0.026), while no significant difference was observed with Juvéderm 24 (3% very much improved, 6% much improved, 40% improved, 50% no change, and 0% worse; *p* > 0.05) [28]. Compared to a untreated control group, findings of a split-face study showed an improvement in GAIS (from ~1.6 to ~1.8) evaluated by investigators in the CaHA-treated side, while an average score of 3.9 corresponding to an unchanged result was observed in the control side. This study concluded an average of very much improved to much improved (1.6 ± 0.70) after treatment that was compared with the control. Also, patients’ evaluations of GAIS were in line with investigators, showing a mean score of 2.3 ± 0.95 correlating to much improved in the CaHA-treated side, while no change was observed in the control group [8]. In a parallel controlled clinical trial, 99.1% of the patients in the CaHA arm showed some level of improvements in GAIS evaluated by investigators (31.9% very much improved, 44% much improved, 23.3% improved, and 0.9% no change). In comparison with the untreated control group, reviewers reported a difference of 39.6% (25.3%, 50.0%) of treatment response of ≥1-point change on both jawlines in CaHA groups compared to the no treatments group. Also, 94% of patients in the CaHA arm reported some level of improvement (27.6% very much improved, 32.8% much improved, 33.6% improved, 5.2% no change, 0.9% worse) [37].

##### Satisfaction

Five out of eight studies reported the findings of satisfaction following injection [8,12,27,28,37]. Findings of a trial comparing CaHA with Juvéderm 24, Perlane, and Juvéderm 24 HV showed significantly higher satisfaction in the CaHA group than the control groups (*p* < 0.001). A total of 97% of patients in the CaHA arm reported some level of satisfaction (39% of participants in the CaHA arm reported extremely satisfied, 51% satisfied, 7% slightly satisfied), with 2% slightly dissatisfied of the CaHA injection and none reported any dissatisfaction [28]. Another trial comparing CaHA with an untreated control group showed significantly higher satisfaction in the CaHA group compared to the control group (*p* < 0.05). Both patients and investigators showed a satisfactory rate at least 75% in CaHA groups compared to the control group [12]. A split-face study comparing CaHA with an untreated control group found high patient satisfaction, without reporting any estimation [8]. In a parallel clinical trial, a higher satisfaction score after the CaHA treatment (mean ± standard deviation (SD) Rasch-transformed scores: 75.2 ± 22.3) compared to the baseline values (mean ± SD Rasch-transformed scores: 21.5 ± 18.9) with the mean difference of 53.9 ± 25.7 was observed; however, no between-group comparison was reported [37]. Findings from a clinical trial examining four dermal fillers, including Aquamid, Sculptra, CaHA, and fat, showed a statistically significant improvement in Aquamid and Sculptra groups (*p* < 0.005), but not in the CaHA and fat arms (*p* > 0.05). Additionally, between-group comparisons indicated significant improvements in Aquamid, Sculptra, and fat groups compared to CaHA (*p* < 0.005) [27]. These studies used either 6-point scale or FACE-Q questionnaires.

##### Wrinkle and Curve Correction

The effects of CaHA on wrinkle and curve/fold/line correction were investigated by four studies [5,8,28,38]. A split-face study examining the effect of CaHA and human-based collagen on nasolabial folds reported a significant within-group improvement (*p* < 0.005) in Lemperle rating scale in both arms. This study also showed that the CaHA-treated sides were superior in approximately 85% of subjects at the end of follow-up, whereas the control sides were superior in only 3% of subjects (*p* < 0.0001) [38]. In comparison with nonanimal hyaluronic acid, another split-face study indicated a greater improvement in wrinkle severity rating scale in 31% of the CaHA-treated nasolabial folds than the hyaluronic acid-treated folds (*p* < 0.0001) [5]. Another split-face study comparing the effect of CaHA with no treatment as the control using Merz jawline scale observed a statistically significant improvement in wrinkles of CaHA-treated area versus control area (*p* < 0.0001). This study reported an average of a 1.3-point improvement in this 5-point assessment tool [8]. A parallel clinical trial examining the effect of CaHA, Juvéderm 24 HV, Juvéderm 24, and Perlane reported no statistically significant differences for any product over any other one according to the wrinkle severity rating scale [28].

#### 3.3.2. Hands

##### Global Aesthetic Improvement

Four studies evaluated global aesthetic improvement using GAIS in dorsum of hands [23,24,25,40]. A comparison between CaHA and hyaluronic acid showed some level of improvements in this score after treatments, but between-groups comparison was provided [40]. Also, another two parallel clinical trials only reported the findings in intervention groups without any comparison with the control groups (no treatment). These studies reported improvements in GAIS in more than 92% of the patients evaluated either by investigators or patients [24,25]. In a parallel clinical trial in comparison with no treatment, between-group comparisons indicated a statistically significant improvement in GAIS score evaluated by investigators in the CaHA group (15.1% very much improved, 45.4% much improved, 30.3% improved, 7.2% no change, and 2% worse) compared to the control group (4% much improved, 8% much improved, 26% improved, 52% no change, and 5% worse; *p* < 0.0001) [23].

##### Hand Grading Scales

All the included studies used hand grading scales to explore the effects of CaHA injection on the hands in comparison with either hyaluronic acid or no treatment [23,24,25,26,40]. A lower Merz hand grading scale (MHGS) score was reported following CaHA intervention by a parallel clinical trial comparing CaHA with hyaluronic acid [40]. At baseline, the mean MHGS score was 3.5 in the CaHA group and 3.4 in the control group, while post-treatment, it decreased to 2.1 in the CaHA group and 1.7 in the control group. No within- or between-groups statistical comparisons were provided, while a split-face study comparing CaHA with hyaluronic acid found a statistically significant increase in MHGS score in the CaHA group relative to the control group (mean change of 1.3 ± 0.7 in CaHA-treated side versus 1.1 ± 0.5 in the control side; *p* = 0.025) [26]. The remaining studies were designed as parallel clinical trials comparing CaHA with no treatments [23,24,25], showing a statistically significant 1-point improvement in MHGS or validated Busso hand volume severity scale in CaHA treatment groups compared to control groups (*p* < 0.05).

##### Other Outcomes

In facial regions; other outcomes, such as subcutaneous thickness [39], assessment of jawline [37], and self-perceived facial damage by lipoatrophy [27], were reported each by one study. Also, in dorsum of hand, one study examined satisfactions [25] and one skin appearance [40] following injection. These findings are presented in Table S3.

## 4. Discussion

The available evidence demonstrates enhancements in GAIS scores subsequent to the injection of CaHA, whether applied in facial regions or the dorsum of hands. The findings show, in general, a high level of satisfaction among patients using CaHA injections, particularly in the facial areas. Additionally, studies indicated improvements in hand grading scales and facial wrinkles also in comparison with the control treatments.

The Food and Drug Administration (FDA) approved the use of CaHA (Radiesse^®^, Merz North America, Inc., Raleigh, NC, USA) for the correction of moderate-to-severe facial folds and wrinkles, such as nasolabial folds, the correction of facial lipoatrophy in patients with human immunodeficiency virus (HIV) infection, jawline contour improvement, and the correction of volume loss in the dorsum of the hands. However, diluted and hyperdiluted calcium hydroxylapatite have also been used for skin tightening [41]. Furthermore, CaHA has been used for different aesthetic purposes in a variety of injection areas, such as jawline, nasolabial folds, orbital region, dorsum of hand and foot, neck, chest, décolletage, and abdomen regions. CaHA gel, with 30% CaHA in 70% carboxymethyl cellulose gel, is an example of regenerative scaffold. Regenerative scaffold is an aesthetic term to describe aesthetic injectable biomaterials that can divert the fibrotic foreign body response toward regeneration [1].

The clinical effectiveness of fillers is influenced by their physiochemical, rheological, and biostimulatory properties, all of which play a role in guiding treatment choices. The distinctive rheological profile of CaHA, characterized by high viscosity and elasticity compared to other fillers, can increase the stiffness of extracellular matrix, causing fibroblast proliferation enhancement and particularly upregulating angiogenic activity [41,42], and establishes a scientific foundation for the tissue support observed during revolumization [43].

CaHA has been employed in diverse medical applications such as vocal cord paralysis, oral surgery, and radiology. Typical adverse responses to CaHA filler comprise immediate redness, swelling, bruising, and occasional lumpiness post-injection. However, it has suggested that fillers might carry the potential risk of foreign body granuloma formation. The incidence rate of true granulomatous reactions to CaHA remains unknown, and published reports of such occurrences are rare [44,45,46]. Notably, among the included studies in the current systematic review, no granulomas, manifestations of allergy, ulcerations, or other serious adverse events were reported. Additionally, in studies conducted on the dorsum of the hands, results from hand function tests indicated no negative impact on hand function as a result of CaHA treatment. The most commonly reported adverse effects in the studies included swelling, hematoma, edema, discomfort/pain, redness, and bruising. Among the studies conducted on the facial area, nodule formation was reported in four studies [5,12,38,39], occurring in one to three participants. In studies focusing on the dorsum of the hand, only one study reported nodule formation in seven subjects (6.2%), and all cases were rated as mild [24].

This review exclusively examines the impact of CaHA on aesthetic outcomes and satisfaction, summarizing the findings of controlled clinical trials. Our study provides a valuable resource for researchers, clinicians, and aesthetic practitioners by presenting the most up-to-date evidence on the effectiveness of this dermal filler in a systematic and thorough manner, adhering to the PRISMA and evidence-based medicine guidelines. Prior published reviews, primarily narrative reviews, lacked a systematic assessment of the evidence, making them susceptible to biases. Additionally, these reviews either did not exclusively focus on CaHA or were often limited to a specific injected area or a specific patient population [47,48,49,50,51,52].

Several limitations were observed in the existing studies, potentially impacting the overall quality of evidence and necessitating caution in interpreting our findings. Methodological concerns, such as a lack of randomization and blinding, were observed in the majority of studies, affecting the validity of their findings. Additionally, some included studies did not conduct appropriate statistical tests to determine the extent of differences, changes, and comparisons within or between intervention and control groups The evaluation methods employed for the majority of the study outcomes were subjective, making them susceptible to bias, which could impact the reliability and consistency of the findings [53]. Therefore, additional clinical trials, are needed to address these limitations to enhance the validity of findings.

Moreover, the present study acknowledges a limitation associated with the heterogeneous comparison groups, presenting a barrier to conducting a meta-analysis. Within the studies encompassed in this review, we observe a diverse range of fillers utilized as comparisons, including Aquamid, Sculptra, fat, poly-L-lactic acid, Juvéderm 24, Juvéderm 24 HV, and Perlane. Each of these fillers was compared in a restricted number of studies, usually a maximum of two. This limitation underscores the complexity in drawing a conclusion concerning the relative impacts of CaHA compared to other treatments, emphasizing the need for careful interpretation and consideration of the study’s outcomes within the context of this inherent heterogeneity. Additionally, controlled clinical trials were only available for facial regions and the dorsum of hands, suggesting that the conduct of studies on body areas beyond the face and hands such as abdomen, décolletage, arms, dorsum of feet, and knee is crucial. Last, factors such as injection depth can have a role in aesthetic outcomes, and this information was not provided precisely by all the included studies in the review. For instance, a recent study revealed that although volumetric fat loss occurs across all layers of the hands with aging, certain layers like the dorsal intermediate lamina exhibit more significant losses. This would suggest that the therapeutic impact of volumetric injection may depend on both the depth of injection and the specific anatomical layer being targeted [54].

## 5. Conclusions

The existing body of evidence suggests that CaHA is effective in enhancing aesthetic outcomes. This is evident through measurements of global aesthetic scores in both facial regions and the dorsum of hands, as well as by the level of the reported satisfaction post-injection in facial areas. Notably, improvements in wrinkles and facial contouring are also noted, along with enhancements in hand grading scales. Given the heterogeneity observed in both comparators and measurement metrics across the included studies, conducting a meta-analysis to quantify the enhancement CaHA compared to other fillers and aesthetic interventions was not feasible. Hence, to comprehensively grasp the clinical effectiveness of CaHA in relation to other established and available fillers, and to explore its full potential for aesthetic treatments also beyond the face and hands, well-designed clinical trials that specifically address the limitations raised in this study are needed.

## Figures and Tables

**Table 1:** Characteristics of the included studies.

Study Characteristics					Participants’ Characteristics				Exposure/Intervention		Outcomes
Author, Year	Country	Design	Comparison	Follow-Up	No. Participants (Int/Cnt) ^1^	Health Characteristics	Sex (%F)	Age ^2^	CaHA Brand, Dosage (Mean)	Injected Area	Outcomes, Assessment Method
**Facial**											
Moers-Carpi, 2008 [5]	Spain, Germany	Split face	Nonanimal stabilized hyaluronic acid	12 m	58 (58/58)	Moderate to severe NLFs	B (86.6)	50.5	Radiesse, 0.88 mL	NLFs	1- Investigators Global Aesthetic Improvement, GAIS. 2- Investigators Wrinkle assessment, WSRS.
Smith, 2007 [38]	US	Split-face	Human-Based Collagen	3 m	115 (115/115)	Moderate to deep NLFs	B (89.74)	54.7 (31–76)	Radiesse, 1.2 (0.3–2.7) mL	NLFs	1- Investigators Wrinkle assessment, LRS. 2- Investigators Correction curves assessment, LRS. 3- Investigators Global Aesthetic Improvement, GAIS.
Moers-Carpi, 2007 [28]	5 European clinic	Parallel	1- Juvéderm 24 2- Juvéderm 24 HV 3- Perlane	12 m	194 (NM/NM)	Moderate to severe NLFs	B (90.24)	52 (27–80)	Radiesse, 2.21 mL	NLFs	1- Patient satisfaction, a 6-point scale. 2- Investigators Wrinkle assessment, WSRS. 3- Investigators Global Aesthetic Improvement, GAIS.
Vallejo, 2017 [27]	Spain	Parallel	1- Aquamid 2- Sculptra 3- Fat	24 m	147 (51/76,14,6)	HIV/facial lipoatrophy	B (18.4)	48 ± 6.9	Radiesse, 29.89 ± 38.46 mL (Per patient)	NLFs, malar, zygomatic, Bichat bulla, temporal, supraorbital	1- Patient satisfaction, a 10-point scale (10 most satisfied). 2- Self-perceived facial damage by lipoatrophy, a 10-point scale (10 most affected).
Boen, 2022 [8]	US	Split- face	No treatment	30 d (control Split remained untreated until day 30)	10 (10/10)	At least Grade 1 (mild) on MJS	F	61.2 (44–69)	Radiesse(+), 3 mL	Jawline	1- Investigator Sagging/ Wrinkling assessment, MJS. 2- Investigators Global Aesthetic Improvement, GAIS. 3- Patients Global Aesthetic Improvement, GAIS. 4- Patient satisfaction, a 5-point scale.
Moradi, 2021 [37]	US	Parallel	No treatment	12 w (control group remained untreated until week 12)	180 (123/57)	Moderate or severe ratings on MJS	B (81.1)	55.3 ± 7.1	Radiesse(+), max limited to 3 mL (Per side)	Jawline	1- Jawline assessment scale, MJS. 2- Investigators Global Aesthetic Improvement, GAIS. 3- Patients Global Aesthetic Improvement, GAIS. 4- Patient satisfaction, FACE-Q.
Rozelaar, 2014 [39]	Netherlands	Parallel	Poly-L-lactic acid	12 m	49 (23/26)	HIV positive patients with combination antiretroviral therapy induced facial lipoatrophy (grades 2–4)	B (6.09)	Control: 51.3 CaHA: 51.2	Radiesse, 9.1 ± 0.6 mL (In multiple sessions, 1.5–3 mL per session)	Buccal and/or Temporal regions	Regional total subcutaneous thickness, MRI.
Moers-Carpi, 2012 [12]	Germany	Parallel	No treatment	12 w (control group remained untreated until week 12)	116 (86/30)	A moderate to severe pretreatment cheeks on a validated cheek volume severity scale	B (97)	56 (27–83)	Radiesse, 4.7 mL (In multiple sessions)	Cheeks (malar, submalar, zygoma, preauricular, infraorbital areas)	1- Patient satisfaction, a 6-point scale. 2- Investigator satisfaction, a 6-point scale.
**Hand**											
Kim, 2019 [40]	Korea	Parallel	Hyaluronic acid	12 m	20 pairs (20 left /20 right)	Soft tissue loss with visible veins and tendons in hands	F	61.8 (45–72)	Radiesse, 1 mL	Dorsum of hand	1- Hand grading, MHGS. 2- Patient Global Aesthetic Improvement, GAIS (0 as worse). 3- Soft tissue loss, Skin roughness, Skin appearance, dermascope.
Sattler, 2014 [26]	Germany	Split- hands	Hyaluronic acid	4–6 w	37 pairs (37/37)	With severe or very severe loss of fatty tissue equivalent to a score of 3 or 4 according to MHGS	F	56 (45–65)	Radiesse, 0.8 mL	Dorsum of hand	Hand grading, MHGS.
Busso, 2010 [23]	Germany	Parallel	No treatment	3 m	101 pairs (76/25)	Right and left hand ratings of 3 or 4 on BHVSS	B (95.05)	Int: 57.4 (37–78) Cnt: 56.9 (43–69)	Radiesse, 3.1 mL (both hands)	Dorsum of hand	1- Hand grading, BHVSS. 2- Investigator Global Aesthetic Improvement, GAIS.
Goldman, 2018 [24]	US	Parallel	No treatment	12 w	114 pairs (85/29)	With a rating of2 or 3 for both hands on the MHGS	B (95.6)	53.3	Merz, 2.6 mL (each hand)	Dorsum of hand	1- Hand grading, MHGS. 2- Patient Global Aesthetic Improvement, GAIS.
Bertucci, 2015 [25]	US	Parallel	No treatment	4 w (control group remained untreated until week 4)	30 pairs (20/10)	Hands with a rating of 2 or 3 in the MHGS	B (93.3)	51.5 (37–65)	Radiesse, 4.5 ± 2.0 mL (both hands)	Dorsum of hand	1- Hand grading, MHGS 2- Investigator Global Aesthetic Improvement, GAIS. 3- Patient Global Aesthetic Improvement, GAIS. 4- Patient satisfaction, 6 point scale. 5- Investigator satisfaction, 6 point scale.

^1^ Intervention group/control group. ^2^ Age is reported either in mean (min, max) or min–max, or mean ± SD. NM: not mentioned; B: both men and women; F: females; NLFs: nasolabial folds; GAIS: Global Aesthetic Improvement Scale; WSRS: Wrinkle Severity Rating Scale; MJS: Merz Jawline Scale; MHGS: Merz Hand Grading Scale; BHVSS: Validated Busso Hand Volume Severity Scale; LRS: Lemperle Rating Scale.

## Data Availability

All data are presendeted in the manuscript and online Supplementary Materials.

## Supplementary Materials

The following supporting information can be downloaded at: https://www.mdpi.com/article/10.3390/jcm13061686/s1. Table S1: Search strategies. Table S2: Risk of bias according to Cochrane Collaboration’s Tool Risk of Bias 2 [5,8,12,23,24,25,26,27,28,37,38,39,40]. Table S3: Summary of additional findings (other outcomes) of the included studies [25,27,37,39,40]. Table S4: Reported assessment methods in facial and hand regions [5,8,12,23,24,25,26,27,28,37,38,39,40].

## References

[1] Corduff N. (2023). Introducing aesthetic regenerative scaffolds: An immunological perspective. J. Cosmet. Dermatol..

[2] (2022). Aesthetic Plastic Surgery National Databank Statistics 2020–2021. Aesthet Surg. J..

[3] AsfAp S. (2020). The aesthetic society’s cosmetic surgery national data bank: Statistics 2019. Aesthetic Surg. J..

[4] Amiri M., Mecani R., Christa N., Phillips T., Janina K., Daughtry C.H., Muka T. (2023). Skin regeneration-related mechanisms of Calcium Hydroxylapatite (CaHA): A systematic review. Front. Med..

[5] Moers-Carpi M.M., Tufet J.O. (2008). Calcium hydroxylapatite versus nonanimal stabilized hyaluronic acid for the correction of nasolabial folds: A 12-month, multicenter, prospective, randomized, controlled, split-face trial. Dermatol. Surg..

[6] Vagefi M.R., McMullan T.F.W., Burroughs J.R., Georgescu D., McCann J.D., Anderson R.L. (2011). Orbital augmentation with injectable calcium hydroxylapatite for correction of postenucleation/evisceration socket syndrome. Ophthalmic Plast. Reconstr. Surg..

[7] Pavicic T., Sattler G., Fischer T., Dirschka T., Kerscher M., Gauglitz G., Dersch H., Kravtsov M., Heide I., Prager W. (2022). Calcium Hydroxyapatite Filler With Integral Lidocaine CaHA (+) for Soft Tissue Augmentation: Results from an Open-Label Multicenter Clinical Study. J. Drugs Dermatol..

[8] Boen M., Alhaddad M., Goldman M.P., Kollipara R., Hoss E., Wu D.C. (2022). A Randomized, Evaluator-Blind, Split-Face Study Evaluating the Safety and Efficacy of Calcium Hydroxylapatite for Jawline Augmentation. Dermatol. Surg..

[9] Rovatti P.P., Pellacani G., Guida S. (2020). Hyperdiluted calcium hydroxylapatite 1:2 for mid and lower facial skin rejuvenation: Efficacy and safety. Dermatol. Surg..

[10] Kim J. (2018). Novel Forehead Augmentation Strategy: Forehead Depression Categorization and Calcium-Hydroxyapatite Filler Delivery after Tumescent Injection. Plast. Reconstr. Surg. Glob. Open.

[11] Grunebaum L.D., Elsaie M.L., Kaufman J. (2010). Six-month, double-blind, randomized, split-face study to compare the efficacy and safety of calcium hydroxylapatite (caha) mixed with lidocaine and caha alone for correction of nasolabial fold wrinkles. Dermatol. Surg..

[12] Moers-Carpi M., Storck R., Howell D.J., Ogilvie P., Ogilvie A. (2012). Physician and patient satisfaction after use of calcium hydroxylapatite for cheek augmentation. Dermatol. Surg..

[13] Wollina U., Goldman A. (2020). Long lasting facial rejuvenation by repeated placement of calcium hydroxylapatite in elderly women. Dermatol. Ther..

[14] Juhász M.L.W., Levin M.K., Marmur E.S. (2018). Pilot Study Examining the Safety and Efficacy of Calcium Hydroxylapatite Filler With Integral Lidocaine Over a 12-Month Period to Correct Temporal Fossa Volume Loss. Dermatol. Surg..

[15] Muti G.F. (2019). Open-Label, Post-Marketing Study to Evaluate the Performance and Safety of Calcium Hydroxylapatite With Integral Lidocaine to Correct Facial Volume Loss. J. Drugs Dermatol..

[16] Aletaha M., Salour H., Yadegary S., Fekri Y., Tavakoli M. (2017). Orbital Volume Augmentation with Calcium Hydroxyapatite Filler in Anophthalmic Enophthalmos. J. Ophthalmic Vis. Res..

[17] Barbarino S.C. (2021). Correction of temporal wasting using calcium hydroxylapatite with integral lidocaine: An underused procedure for enhancing overall facial appearance. J. Cosmet. Dermatol..

[18] Becker H. (2008). Nasal augmentation with calcium hydroxylapatite in a carrier-based gel. Plast. Reconstr. Surg..

[19] Carruthers A., Carruthers J. (2008). Evaluation of injectable calcium hydroxylapatite for the treatment of facial lipoatrophy associated with human immunodeficiency virus. Dermatol. Surg..

[20] Corduff N. (2020). An Alternative Periorbital Treatment Option Using Calcium Hydroxyapatite for Hyperpigmentation Associated with the Tear Trough Deformity. Plast. Reconstr. Surg. Glob. Open.

[21] Juhász M.L.W., Marmur E.S. (2018). Examining the Efficacy of Calcium Hydroxylapatite Filler with Integral Lidocaine in Correcting Volume Loss of the Jawline-A Pilot Study. Dermatol. Surg..

[22] Tzikas T.L. (2004). Evaluation of the Radiance FN soft tissue filler for facial soft tissue augmentation. Arch. Facial Plast. Surg..

[23] Busso M., Moers-Carpi M., Storck R., Ogilvie P., Ogilvie A. (2010). Multicenter, randomized trial assessing the effectiveness and safety of calcium hydroxylapatite for hand rejuvenation. Dermatol. Surg..

[24] Goldman M.P., Moradi A., Gold M.H., Friedmann D.P., Alizadeh K., Adelglass J.M., Katz B.E. (2018). Calcium Hydroxylapatite Dermal Filler for Treatment of Dorsal Hand Volume Loss: Results From a 12-Month, Multicenter, Randomized, Blinded Trial. Dermatol. Surg..

[25] Bertucci V., Solish N., Wong M., Howell M. (2015). Evaluation of the Merz Hand Grading Scale After Calcium Hydroxylapatite Hand Treatment. Dermatol. Surg..

[26] Sattler G., Walker T., Buxmeyer B., Biwer B. (2014). Efficacy of calcium hydroxylapatite filler versus hyaluronic acid filler in hand augmentation. Aktuel Dermatol..

[27] Vallejo A., Garcia-Ruano A.A., Pinilla C., Castellano M., Deleyto E., Perez-Cano R. (2018). Comparing Efficacy and Costs of Four Facial Fillers in Human Immunodeficiency Virus-Associated Lipodystrophy: A Clinical Trial. Plast. Reconstr. Surg..

[28] Moers-Carpi M., Vogt S., Santos B.M., Planas J., Vallve S.R., Howell D.J. (2007). A multicenter, randomized trial comparing calcium hydroxylapatite to two hyaluronic acids for treatment of nasolabial folds. Dermatol. Surg..

[29] Moher D., Liberati A., Tetzlaff J., Altman D.G., Group P. (2009). Preferred reporting items for systematic reviews and meta-analyses: The PRISMA statement. BMJ.

[30] Muka T., Glisic M., Milic J., Verhoog S., Bohlius J., Bramer W., Chowdhury R., Franco O.H. (2020). A 24-step guide on how to design, conduct, and successfully publish a systematic review and meta-analysis in medical research. Eur. J. Epidemiol..

[31] Sterne J.A.C., Savović J., Page M.J., Elbers R.G., Blencowe N.S., Boutron I., Cates C.J., Cheng H.Y., Corbett M.S., Eldridge S.M. (2019). RoB 2: A revised tool for assessing risk of bias in randomised trials. BMJ.

[32] Figueredo V.O., Miot H.A., Soares Dias J., Nunes G.J.B., Barros de Souza M., Bagatin E. (2020). Efficacy and Safety of 2 Injection Techniques for Hand Biostimulatory Treatment With Diluted Calcium Hydroxylapatite. Dermatol. Surg..

[33] Gubanova E.I., Starovatova P.A. (2015). A Prospective, Comparative, Evaluator-blind Clinical Study Investigating Efficacy and Safety of Two Injection Techniques with Radiesse(R) for the Correction of Skin Changes in Aging Hands. J. Cutan. Aesthet. Surg..

[34] Wu D.C., Goldman M.P. (2018). Randomized, Double-Blinded, Sham-Controlled, Split-Hand Trial Evaluating the Safety and Efficacy of Triamcinolone Acetate Injection after Calcium Hydroxylapatite Volume Restoration of the Dorsal Hand. Dermatol. Surg..

[35] Marmur E., Green L., Busso M. (2010). Controlled, randomized study of pain levels in subjects treated with calcium hydroxylapatite premixed with lidocaine for correction of nasolabial folds. Dermatol. Surg..

[36] Godin M.S., Majmundar M.V., Chrzanowski D.S., Dodson K.M. (2006). Use of radiesse in combination with restylane for facial augmentation. Arch. Facial Plast. Surg..

[37] Moradi A., Green J., Cohen J., Joseph J., Dakovic R., Odena G., Verma A., Scher R. (2021). Effectiveness and Safety of Calcium Hydroxylapatite With Lidocaine for Improving Jawline Contour. J. Drugs Dermatol..

[38] Smith S., Busso M., McClaren M., Bass L.S. (2007). A randomized, bilateral, prospective comparison of calcium hydroxylapatite microspheres versus human-based collagen for the correction of nasolabial folds. Dermatol. Surg..

[39] Van Rozelaar L., Kadouch J.A., Duyndam D.A., Nieuwkerk P.T., Lutgendorff F., Karim R.B. (2014). Semipermanent filler treatment of hiv-positive patients with facial lipoatrophy: Long-term follow-up evaluating MR imaging and quality of life. Aesthet. Surg. J..

[40] Kim J.S. (2019). Detailed Sonographic Anatomy of Dorsal Hand Augmentation with Hyaluronic Acid and Calcium Hydroxyapatite Fillers. Aesthet. Surg. J..

[41] Goldie K. (2023). The evolving field of regenerative aesthetics. J. Cosmet. Dermatol..

[42] Ali M., Khan N.R., Basit H.M., Mahmood S. (2020). Physico-chemical based mechanistic insight into surfactant modulated sodium Carboxymethylcellulose film for skin tissue regeneration applications. J. Polym. Res..

[43] Lorenc Z.P., Bass L.M., Fitzgerald R., Goldberg D.J., Graivier M.H. (2018). Physiochemical characteristics of calcium hydroxylapatite (CaHA). Aesthetic Surg. J..

[44] Csuka D.A., Ha J., Hanna A.S., Kim J., Phan W., Ahmed A.S., Ghoniem G.M. (2023). Foreign body granuloma development after calcium hydroxylapatite injection for stress urinary incontinence: A literature review and case report. Arab. J. Urol..

[45] Lemperle G., Gauthier-Hazan N. (2009). Foreign body granulomas after all injectable dermal fillers: Part 2. Treatment options. Plast. Reconstr. Surg..

[46] Sankar V., McGuff H.S. (2007). Foreign body reaction to calcium hydroxylapatite after lip augmentation. J. Am. Dent. Assoc..

[47] Trinh L.N., Gupta A. (2021). Non-Hyaluronic Acid Fillers for Midface Augmentation: A Systematic Review. Facial Plast. Surg..

[48] Sturm L.P., Cooter R.D., Mutimer K.L., Graham J.C., Maddern G.J. (2011). A systematic review of dermal fillers for age-related lines and wrinkles. Anz. J. Surg..

[49] Ovadia S.A., Efimenko I.V., Lessard A.S. (2021). Dorsal Hand Rejuvenation: A Systematic Review of the Literature. Aesthetic Plast. Surg..

[50] McGuire C., Boudreau C., Tang D. (2022). Hand Rejuvenation: A Systematic Review of Techniques, Outcomes, and Complications. Aesthetic Plast. Surg..

[51] Jagdeo J., Ho D., Lo A., Carruthers A. (2015). A systematic review of filler agents for aesthetic treatment of HIV facial lipoatrophy (FLA). J. Am. Acad. Dermatol..

[52] Fakhre G.P., Perdikis G., Shaddix K.K., Terkonda S.P., Waldorf J.C. (2009). An evaluation of calcium hydroxylapatite (Radiesse) for cosmetic nasolabial fold correction: A meta-analysis and patient centric outcomes study. Ann. Plast. Surg..

[53] Adams A.S., Soumerai S.B., Lomas J., Ross-Degnan D. (1999). Evidence of self-report bias in assessing adherence to guidelines. Int. J. Qual. Health Care.

[54] Hung Y.T., Cheng C.Y., Chen C.B., Huang Y.L. (2022). Ultrasound Analyses of the Dorsal Hands for Volumetric Rejuvenation. Aesthet. Surg. J..

