# Supplementary material for: Calcium Hydroxylapatite (CaHA) and Aesthetic Outcomes: A Systematic Review of Controlled Clinical Trials
Source: J Clin Med. 2024 Mar 14;13(6):1686. doi: 10.3390/jcm13061686 (PMC10971119; doi:10.3390/jcm13061686)
Supplement: Supplementary file 1 [file jcm-13-01686-s001.zip › jcm-2839039-supplementary/jcm-2839039-supplementary.pdf]

Supplementary files: Calcium hydroxylapatite (CaHA) and aesthetic outcomes: a systematic review of controlled clinical trials

Table S1. Search strategies.

**Database**	**Strategy**
**Embase**	((radiesse OR CaHA OR calcium-hydroxyapatit* OR calcium-hydroxylapatit*):ab,ti,kw) NOT ([Conference Abstract]/lim OR 'letter'/de OR 'note'/de OR 'review'/exp OR (systematic-review* OR meta-analys* OR metaanalys*):ti)
**Medline**	((radiesse OR CaHA OR calcium-hydroxyapatit* OR calcium-hydroxylapatit*).ab,ti,kf.) NOT ((news OR congres* OR abstract* OR book* OR chapter* OR dissertation abstract*).pt. OR (systematic-review* OR meta-analys* OR metaanalys*).ti.)
**Web of Science**	TS=((radiesse OR CaHA OR calcium-hydroxyapatit* OR calcium-hydroxylapatit*)) **NOT** TI=((systematic-review* OR meta-analys* OR metaanalys*)) **AND** DT=(Article OR Review OR Letter OR Early Access)
**Cochrane**	((radiesse OR CaHA OR calcium-hydroxyapatit* OR calcium-hydroxylapatit*):ab,ti,kw) NOT ((systematic-review* OR meta-analys* OR metaanalys*):ti)
**Google Scholar**	radiesse|CaHA|"calcium hydroxyapatite"|"calcium hydroxylapatite" -orbital|patulous|Eustachian|vocal|dental|teeth|tooth|voice|osteoporose|osteomyelitis|bone|scars

Table S2. Risk of bias according to Cochrane Collaboration's Tool Risk of Bias 2.

Author, year	Bias arising from					Overall score
	**Randomization process**	**Intended interventions**	**Missing data**	**Outcome measurement**	**Selection of the reported result**	
Moers-Carpi, 2008 (1)	Some concerns	High risk	Low risk	Some concerns	Low risk	High risk
Smith, 2007 (2)	High risk	High risk	Low risk	Low risk	Low risk	High risk
Moers-Carpi, 2007 (3)	Some concerns	High risk	Low risk	Low risk	Low risk	High risk
Vallejo, 2017 (4)	High risk	High risk	Low risk	High risk	Low risk	High risk
Boen, 2022 (5)	Some concerns	High risk	Low risk	Some concerns	Low risk	High risk
Moradi, 2021 (6)	Some concerns	High risk	Low risk	Some concerns	Low risk	High risk
Rozelaar, 2014 (7)	High risk	High risk	High risk	Some concerns	Low risk	High risk
Moers-Carpi, 2012 (8)	Low risk	Low risk	Low risk	High risk	Low risk	High risk
Kim, 2019 (9)	High risk	High risk	Low risk	Some concerns	Low risk	High risk
Sattler, 2014 (10)	Some concerns	High risk	Low risk	Low risk	Low risk	High risk
Busso, 2010 (11)	Some concerns	High risk	Low risk	Some concerns	Low risk	High risk
Goldman, 2018 (12)	Low risk	Low risk	Low risk	High risk	Low risk	High risk
Bertucci, 2015 (13)	Low risk	Low risk	Low risk	High risk	Low risk	High risk

Table S3. Summary of additional findings (Other outcomes) of the included studies

Author, year	Design	Comparison	Injected area	Results
**Facial**				
Vallejo, 2017 (4)	Parallel	1- Aquamid<br>2- Sculptra<br>3- Fat	Multi areas	**Self-Perceived Facial Damage:**<br>*CaHA - Post vs Pre*: p<0.001.<br>*Aquamid - Post vs Pre:* p<0.001.<br>*Aquamid vs CaHA*: p<0.001.<br>*Sculptra - Post vs Pre:* p= 0.002.<br>*Sculptra vs CaHA*: p= 0.001.<br>*Fat - Post vs Pre:* p=0.034.<br>*Fat vs CaHA*: p<0.001.
Moradi, 2021 (6)	Parallel	No treatment	Jawline	**Jawline assessment scale**:<br>*Post-CaHA*: 75.6% showed a ≥ 1-point improvement on the MJAS in both jawlines compared to baseline. The responder rates were significantly greater than 50% (p<0.0001), meeting the threshold for clinical effectiveness.<br>*Post-control*: only 8.8% of patients in the control group were responders.<br>*CaHA vs control*: A statistically significant difference of 66.8% (p<0.0001) was demonstrated between the response rates (95%CI:3.7%, 75.2%).
Rozelaar, 2014 (7)	Parallel	Poly-L-lactic acid	Buccal and/or Temporal regions	**Regional total subcutaneous thickness**:<br>*Pre-CaHA (Mean±SE)*:<br>Buccal: 9.3±0.6<br>Temporal: 4.7±0.5<br>*Post-CaHA (Mean±SE)*:<br>Buccal: 12.5±0.6<br>Temporal: 5.6±0.5<br>*Post vs Pre (Mean±SE)*:<br>Buccal: 3.1±0.4<br>Temporal: 0.9±0.4<br>*Pre-Control (Mean±SE)*:<br>Buccal: 9.3±0.6<br>Temporal: 4.5±0.6<br>*Post-Control (Mean±SE)*:<br>Buccal: 12.8±0.5<br>Temporal: 6.7±0.7<br>*Post vs Pre (Mean±SE)*:<br>Buccal: 3.6±0.3<br>Temporal: 2.2±0.4<br>*CaHA vs Control*:<br>Buccal: p=0.46

Author, year	Design	Comparison	Injected area	Results
				Temporal: p=0.03
**Hands**				
Kim, 2019 (9)	Parallel	Hyaluronic acid	Dorsum of hand	**Soft tissue loss, Skin roughness, and Skin appearance**: Soft tissue losses in hands improved and the veins and tendons became less apparent after injections. The skin roughness and appearance improved in both sets of hands on dermascope.
Bertucci, 2015 (13)	Parallel	No treatment	Dorsal hand	**Patient satisfaction:** extremely satisfied (13/20; 65%) or satisfied (3/20; 15%).<br>**Investigator satisfaction:** extremely satisfied (15/20; 75%) or satisfied (4/20; 20%) with treatment outcomes.

NLFs: Nasolabial folds

Table S4. Reported assessment methods in facial and hand regions.

Outcome	Assessment method	Scoring system	Studies
**Facial**			
Global Aesthetic Improvement	GAIS	5 or 7 point scale; either as “very much improved, much improved, improved, no change, worse” or “much improved, moderately improved, minimally improved, no change, minimally worse, moderately worse, much worse” or “very much improved, much improved, improved, no change, worse, much worse, very much worse”.	Moers-Carpi, 2008 (1); Smith, 2007 (2); Moers-Carpi, 2007 (3).
Satisfaction	6-point scoring system	6 as extremely satisfied, 5 as satisfied, 4 as slightly satisfied, 3 as slightly dissatisfied, 2 as dissatisfied, 1 as extremely dissatisfied.	Moers-Carpi, 2007 (3); Moers-Carpi, 2012 (8)
	10-point scoring system	10 as the most satisfied and 0 as the least satisfied.	Vallejo, 2017 (4)
	FACE-Q	NM	Moradi, 2021 (6)
Wrinkle/ Curve/ fold correction	WSRS	5 point scale; 1 as no visible folds to 5 as severe folds.	Moers-Carpi, 2008 (1); Moers-Carpi, 2007 (3)
	LRS	5 point scale; 0 as no wrinkles, 1 as just perceptible wrinkle, 2 as shallow wrinkle, 3 as moderately deep wrinkle, 4 as deep wrinkle, well defined edges, 5 as very deep wrinkle, redundant fold.	Smith, 2007 (2)
	MJS	NM	Boen, 2022 (5)
Thickness	MRI	-	Rozelaar, 2014 (7)
**Hand**			
Hand grading	MHGS	4-point scale; 0 as no loss of fatty tissue, 1 as mild loss/slight visible veins, 2 as moderate loss/mild visible veins and tendons, 3 as severe loss/moderate visible	Kim, 2019 (9); Sattler, 2014 (10); Goldman, 2018 (12); Bertucci, 2015 (13)

Outcome	Assessment method	Scoring system	Studies
		veins, 4 as very severe loss/marked visible veins and tendons.	
	BHVSS		Busso, 2010 (11);
Satisfaction	6-point scale	NM	Bertucci, 2015 (13)
Global Aesthetic Improvement	GAIS	5 or 7 point scale; either as “very much improved, much improved, improved, no change, worse” or “exceptional improvement, very improved, improved, unaltered, worsened” or “very much improved, much improved, improved, no change, worse, much worse, very much worse” or “very much improved to worse than the original condition”.	Kim, 2019 (9); Goldman, 2018 (12); Bertucci, 2015 (13)

GAIS: Global Aesthetic Improvement Scale; WSRS: Wrinkle Severity Rating Scale; MNLFS: Merz Nasolabial Folds Scale; MMLS: Merz Marionette Lines Scale; MJS: Merz Jawline Scale; MHGS: Merz Hand Grading Scale; BHVSS: Validated Busso Hand Volume Severity Scale; LRS: Lemperle Rating Scale; NM: Not mentioned.

REFRENCES:

1. Moers-Carpi MM, Tufet JO. Calcium hydroxylapatite versus nonanimal stabilized hyaluronic acid for the correction of nasolabial folds: A 12-month, multicenter, prospective, randomized, controlled, split-face trial. Dermatol Surg. 2008;34(2):210-5.
2. Smith S, Busso M, McClaren M, Bass LS. A randomized, bilateral, prospective comparison of calcium hydroxylapatite microspheres versus human-based collagen for the correction of nasolabial folds. Dermatol Surg. 2007;33(SUPPL. 2):S112-S21.
3. Moers-Carpi M, Vogt S, Santos BM, Planas J, Vallve SR, Howell DJ. A multicenter, randomized trial comparing calcium hydroxylapatite to two hyaluronic acids for treatment of nasolabial folds. Dermatol Surg. 2007;33(SUPPL. 2):S144-S51.
4. Vallejo A, Garcia-Ruano AA, Pinilla C, Castellano M, Deleyto E, Perez-Cano R. Comparing Efficacy and Costs of Four Facial Fillers in Human Immunodeficiency Virus-Associated Lipodystrophy: A Clinical Trial. Plast Reconstr Surg. 2018;141(3):613-23.
5. Boen M, Alhaddad M, Goldman MP, Kollipara R, Hoss E, Wu DC. A Randomized, Evaluator-Blind, Split-Face Study Evaluating the Safety and Efficacy of Calcium Hydroxylapatite for Jawline Augmentation. Dermatol Surg. 2022;48(1):76-81.
6. Moradi A, Green J, Cohen J, Joseph J, Dakovic R, Odena G, et al. Effectiveness and Safety of Calcium Hydroxylapatite With Lidocaine for Improving Jawline Contour. J Drugs Dermatol. 2021;20(11):1231-8.
7. Van Rozelaar L, Kadouch JA, Duyndam DA, Nieuwkerk PT, Lutgendorff F, Karim RB. Semipermanent filler treatment of hiv-positive patients with facial lipoatrophy: Long-term follow-up evaluating MR imaging and quality of life. Aesthet Surg J. 2014;34(1):118-32.
8. Moers-Carpi M, Storck R, Howell DJ, Ogilvie P, Ogilvie A. Physician and patient satisfaction after use of calcium hydroxylapatite for cheek augmentation. Dermatol Surg. 2012;38(7 PART 2):1217-22.
9. Kim JS. Detailed Sonographic Anatomy of Dorsal Hand Augmentation with Hyaluronic Acid and Calcium Hydroxyapatite Fillers. Aesthet Surg J. 2019;39(10):1096-106.
10. Sattler G, Walker T, Buxmeyer B, Biwer B. Efficacy of calcium hydroxylapatite filler versus hyaluronic acid filler in hand augmentation. Aktuel Dermatol. 2014;40(11):445-51.
11. Busso M, Moers-Carpi M, Storck R, Ogilvie P, Ogilvie A. Multicenter, randomized trial assessing the effectiveness and safety of calcium hydroxylapatite for hand rejuvenation. Dermatol Surg. 2010;36(SUPPL. 1):790-7.
12. Goldman MP, Moradi A, Gold MH, Friedmann DP, Alizadeh K, Adelglass JM, Katz BE. Calcium Hydroxylapatite Dermal Filler for Treatment of Dorsal Hand Volume Loss: Results From a 12-Month, Multicenter, Randomized, Blinded Trial. Dermatol Surg. 2018;44(1):75-83.
13. Bertucci V, Solish N, Wong M, Howell M. Evaluation of the Merz Hand Grading Scale After Calcium Hydroxylapatite Hand Treatment. Dermatol Surg. 2015;41:S389-S96.
